# Dry needling on latent and active myofascial trigger points versus oral diclofenac in patients with knee osteoarthritis: a randomized controlled trial

**DOI:** 10.1186/s12891-022-06116-9

**Published:** 2023-01-18

**Authors:** Yan-Tao Ma, Yu-Lin Dong, Bo Wang, Wen-Pin Xie, Qiang-Min Huang, Yong-Jun Zheng

**Affiliations:** 1 Department of Pain Management, Huadong Hospital Affiliated to Fudan University, Shanghai, China; 2 Department of Treatment, Yang Zhi Affiliated Rehabilition Hospital of Tongji, Shanghai, China; 3 Department of Sport Rehabilitation, School of Kinesiology, Shanghai University of Sport, Shanghai, China; 4 Department of Pain Management, Shanghai Ciyuan Rehablitation Hospital, Sinophama Holding, Shanghai, China

**Keywords:** Knee osteoarthritis, Myofascial trigger points, Dry needling, Stretching, Diclofenac

## Abstract

**Background:**

Latent and active myofascial trigger points (MTrPs) in knee-associated muscles may play a key role in pain management among patients with knee osteoarthritis (KOA). The aim of this study was to investigate the effect of dry needling treatment on pain intensity, disability, and range of motion (ROM) in patients with KOA.

**Methods:**

This randomized, single-blinded, clinical trial was carried out for 6 weeks of treatment and 6-month follow-up. A total of 98 patients met the entry criteria and were randomly assigned to the dry needling latent and active myofascial trigger point (MTrPs) with the stretching group or the oral diclofenacwith the stretching group. Numeric Pain Rating Scale (NPRS), Western Ontario and McMaster Universities Osteoarthritis Index (WOMAC), and ROM were statistically analyzed before and after treatment and at the 6-month follow-up.

**Results:**

A total of 42 patients in the dry needling group (DNG) and 35 patients in the diclofenac group (DG), respectively, completed the study, and there was no significant difference in the general data between the two groups. After treatments, both the groups showed a good effect in knee pain, function, and ROM, However, the DNG showed a significantly better result than the DG. Especially in the results of the 6-month follow-up, the DNG showed much better results than the DG.

**Conclusions:**

Dry needling on latent and active MTrPs combined with stretching and oral diclofenac combined with stretching can effectively relieve pain, improve function, and restore knee ROM affected by KOA. However, the effects of dry needling and stretching are better and longer lasting than those of oral diclofenac and stretching for at least 6 months.

**Trial registration:**

Registered in the Chinese Clinical Trial Registry (www.chictr.org.cn) in 17/11/2017 with the following code: ChiCTR-INR-17013432.

## Background

Osteoarthritis of the knee is a chronic degenerative articular disease that often affects the elderly [1]. The pathological mechanism of knee osteoarthritis (KOA) involves destruction of the articular cartilage and the subchondral bone and gradual irritation of the joint synovium and ligaments with varying degrees of inflammatory changes and joint effusion [2]. Pain is the main clinical manifestation in patients with KOA and is the main reason to seek medical attention. In severe cases, a considerable reduction in knee joint mobility, stiffness, and limited function markedly influences a patient’s quality of life [3].

There are currently no disease-modifying therapies, and therefore, the goal of treatment is to treat KOA-related symptoms, including pain and stiffness, with the goal of minimizing dysfunction and improving the quality of life. Some solutions for treating this disease include lifestyle changes, medications, physical therapy, and finally surgical interventions [4]. Among these methods, drug intervention is still the most commonly used prescription treatment for medical service providers and the most commonly used treatment for KOA patients, for example, oral non-steroidal anti-inflammatory drugs (NSAIDs) such as diclofenac; however, the associated gastrointestinal, renal, and cardiovascular adverse effects of these agents can potentially limit their long-term use [3]. Therefore, studies have shown an increased tendency of KOA patients to use complementary and alternative treatments [5, 6], such as patient education, exercise, weight loss, and physical therapy, which can provide substantial benefits. However, the effect of exercise treatment decreases over time, and self-management education programs result in no or small benefits in people with KOA [7].Currently, radiographs remain the usual means fortheassessment of osteoarthritic changes in the knee and their association with clinical features, mainly based on the Kellgren − Lawrence grade. However, many studies have shown a disagreement between common radiographic findings and clinical symptoms; it is estimated that up to 40% of patients with radiologic damage do not report pain [8], even after total knee arthroplasty(TKA), and between 15 and 20% of patients are not satisfied with the results of the operation, with the main cause of dissatisfaction being pain [9].

The pain of KOA may not be just arthrogenic; it may result from other factors. Myofascial trigger points (MTrPs) are considered hyperirritable spots in a taut band of skeletal muscle, which may produce pain, muscle weakness, and decreased ROM symptoms, depending on their relationship with symptoms [10]. It is classified as active or latent according to its relationship to symptoms. The difference between active and latent MTrPs is that active MTrPs reproduce the pain symptoms experienced by an individual [11], while latent MTrPs can be present without spontaneous symptoms, and when elicited, they do not reproduce the symptoms of an individual. However, latent MTrPs can induce motor dysfunctions, such as stiffness, restriction of range of motion, and muscle fatigue, supporting their clinical relevance.MTrPs have been shown to be related to various chronic skeletal muscle pains, such as neck and shoulder pain [12], low back pain [13], and chronic pelvic pain [14], thatare also found to be associated with KOA. The prevalence of the MTrPs varied from 11 to 50% in different muscles of patients with mild to moderate painful KOA [10] and a prevalence approaching 100% in patients in patients waitlisted for total knee arthroplasty, especially in the medial gastrocnemius and vastus medialis muscles [15].The results of a few existing studies on the treatment of KOA by dry needling are very positive in terms of the improvementsin pain and function [16, 17]. In contrast, a recent systematic review about the effects of dry needling on the MTrPs in patients with knee pain syndromes revealed that this approach was effective for decreasing pain in patellofemoral pain, but was not in knee OA [18]. In fact, only two articles on dry needling for KOA were included in this study, and these studies had one thing in common: the targets of dry needling were all active MTrPs, and no latent MTrPs were involved. Indeed, the epidemiology of latent MTrPs shows a higher prevalence in KOA than active MTrPs [10]. It remains unclear whether latent MTrPs play an important role in KOA, and whether treatment of latent and active MTrPs is effective in KOA.

This study aimed at determining the clinical effects of dry needling on latent and active MTrPs with stretching exercise for KOA and compared with the effect of oral diclofenac with stretching exercise over a 6-week treatment course. We hypothesized that dry needling on latent and active MTrPs combined with stretching can better improve the symptoms and functional abilities of patients with KOA.

## Methods

### Study design

A single-blind trial with 6-month of follow-up with parallel groups was carried out from November 2017 to June 2019. All of the procedures were reviewed and approved by the research ethics committee of Shanghai University of Sport Institute (Approval code: 2,017,017), and this study was carried out in accordance with the World Medical Association’s Declaration of Helsinki (1964). This randomized controlled trial (RCT) has been registered at the Primary Registry of International Clinical Trial Registry Platform World Health Organization “Chinese Clinical Trial Registry” [ChiCTR-INR-17013432], It should be noted that a small change was made after registration. The inclusion of WOMAC is the result of evaluating knee symptoms and functions.

### Sample size

There are no previous studies on the trigger point compared with oral diclofenac for KOA. For this reason, we did not use data from the literature to calculate the sample size. Based on previous pilot trials, the primary outcome measures Numeric Pain Rating Scale (NPRS) and Western Ontario and McMaster Universities Osteoarthritis Index (WOMAC) were used to estimate the sample size, assuming an effect size of 0.4, using G Power 3.1.9.4, assuming a two-tailed test, an alpha level (α) of 0.05, and a desired power (β) of 90%. The estimated desired sample size was calculated to be at least 34 subjects per group. To compensate for a 20% drop-out rate, the sample size was increased to 41 patients in each group.

### Participants

**Fig. 1:**
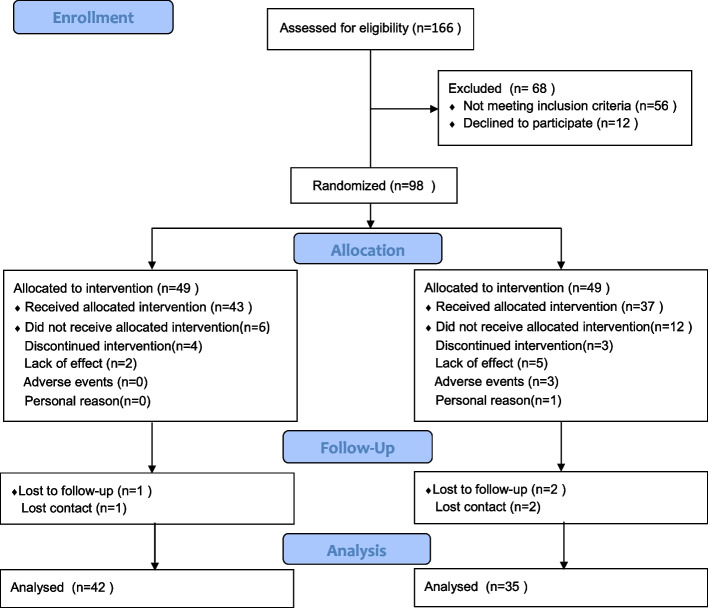
Flowchart of the study participants

A total of 166 participants were assessed for eligibility. Among them, 98 patients with KOA confirmed by radiographic analysis were recruited from the Rehabilitation Department in Shangti Injury Orthopedic and Department of Pain Management, Huadong Hospital affiliated to Fudan University, All of the patients refused to undergo any surgery and signed the informed consent forms. The CONSORT flow diagram for the RCT to study the effects of dry needling on MTrPs versus oral diclofenac in patients with KOA is illustrated in Fig. 1.

To be eligible, participants had to satisfy the following clinical criteria:The inclusion criteria were participants aged 55 years or older with knee pain and uni- or bilateral dysfunction, primary KOA fulfilling the American College of Rheumatology criteria for clinical and Kellgren-Lawrence grades II, III, and IV upon radiographic classification [19, 20]. An average knee pain severity over the past week of ≥ 4 of 10 on an 11-point NPRS and at least one active or one latent MTrP elicited by palpation ipsilateral to the painful knee(s) that was situated in a taut band of a skeletal muscle of the lower limb(s) that usually leads to pain. The exclusion criteria were previous knee surgeries, knee steroid injections within 2 months before the study, or administration of other steroids within 4 weeks before the study. Patients were excluded from the study if they suffered from other conditions that could cause myofascial or neuropathic pain in the lower limb, such as lumbar radiculopathy, saphenous nerve entrapment, and meralgia paresthetica; history of peptic ulcer, heart disease, kidney failure, and known hypersensitivity to diclofenac and other organ-related issues and psychosis; history of metal allergy or needle phobia; advanced osteoporosis, inability to walk 10 m without an assistive device, and inability to comprehend and complete study assessments [21].

### Randomization and blinding

A total of 98 patients who fulfilled the inclusion criteria were randomly assigned (1:1 ratio) to the two dry needling group (DNG) or the control group (diclofenac group) by an independent researcher using Microsoft Excel 2010 via computerized random numbers [22]. An independent researcher, who was not involved in the recruitment, assessment, or intervention process, conducted randomization and was blinded to group allocation. Participants were also instructed not to reveal their group. Sequentially numbered sealed envelopes were used for allocation. The envelopes were only opened by the researcher responsible for applying the treatment programs.

### Outcome measurement

Outcome measures were assessed at baseline, 6 weeks,and 6 months by the same research assistants who had previously been blinded to intervention allocation. Demographic variables were gender, age, height, weight, body mass index (BMI), Duration of pain, Kellgren-Lawrence OA grade and Prevalence of active and latent MTrPs.

### Primary outcome measures

The NPRS was used to measure knee pain intensity. Patients were asked to indicate the average intensity of knee pain over the past 24 h. using an 11-point scale ranging from 0 (“no pain”) to 10 (“worst pain imaginable”) at baseline, 2 weeks, 6 weeks, and 6 months following the initial treatment session. The NPRS is a reliable and valid instrument to assess pain intensity [23].

### Secondary outcome measures

Active range of motion (ROM) of knee flexion was recorded using a long-arm goniometer. The participant was placed in the prone position and was asked to bring the heel of the tested leg as close as possible to the buttock with the contralateral lower limb in extension. The goniometer’s pivot tip was placed on the femur’s lateral epicondyle of the tested knee with one arm in line with the lateral malleolus and the other arm in line with the greater trochanter, The fully extended knee was considered to be the zero position, and the degree of maximum flexion was recorded. The degree of maximum flexion recorded has been shown to be a reliable and valid measure in clinical practice [24].

The WOMAC is the most widely used instrument to evaluate the symptomatology and function in KOA. It contains 24 questions: 5 questions about pain (0–20 points), 2 questions about stiffness (0–8 points), and 17 questions about physical function difficulty (0–68 points), which can be completed in less than 5 min. Higher WOMAC scores (WOMAC pain, WOMAC stiffness, and WOMAC physical function) indicate a greater degree of deterioration [25, 26].

### Interventions

#### The dry needling group (DNG)

##### Examination and location of MTrPs

The diagnosis of active and latent MTrPs followed the criteria described by the International Delphi Panel [11, 27]. 1, palpable taut band; 2, hypersensitive spot on the taut band; and 3,palpation with the reproduction of the patient’s symptoms that include different sensory sensations, including pain spreading to a distant area, deep pain, dull ache, tingling, or burning pain. If the above three points are satisfied, it is considered to be an active trigger point, and if both 1 and 2 are met, but not 3, then it is considered a latent trigger point. The quadriceps (rectus femoris, vastus medialis, and vastus lateralis), tensor fasciae latae, hip adductors(,adductor longus, adductor brevis), hip abductors (gluteus medius, gluteus maximus), hamstrings(biceps femoris, semitendinosus, semimembranosus),triceps calf (Gastrocnemius, Soleus), and popliteus muscles were examined in each subject following a protocol regarding patient and limb positions reproduced from the study of Sánchez et al. [10] and Dor et al. [28] These muscles are frequently involved in myofascial knee pain. When a trigger point is diagnosed, it is marked with a surgical marker. To evaluate the position next time, the patient is required not to clean the marker. Diagnostic criteria were applied by physical therapists with five years of experience with myofascial pain.

##### Methods of treatments

After the skin was locally sterilized, the MTrPs were transcutaneously punctured through the marks with a 0.30 mm × 40 mm or 0.30 mm × 75 mm sterilized disposable stainless-steel acupuncture needle. During dry needling, the left thumb or two fingers pressed or held the taut band. The right hand was used to quickly insert the needle into the entire layer of the skin to avoid skin tingling. The needle tip was then punctured back and forth into the taut band in different directions until a local twitch response was elicited. Once the first local twitch response was obtained, the needle was moved up and down (3–5-mm vertical motions with no rotations) at approximately 1 Hz until no more local twitch responses were elicited; if the patient's tolerance was poor, 1 or 2 local twitch responses were sufficient. The depth of needle insertion ranged from 15 to 65 mm and it depended on the depth of the muscles selected and the thickness of the fat. After pulling out the needle, ischemic compression with a finger was applied for 10 − 30 s. Dry needling treatment was administered once a week and dry needling treatments were terminated once VAS score was less than 3. For the trigger point marked by the surgical marker, if there is only a taut band but no hypersensitive spot, and no palpation with the reproduction of the patient’s symptoms at the next examination, the treatment of the trigger point will be terminated. In addition, the patients were taught to perform self-stretching exercises of knee flexors and extensors at home.

Knee flexor self-stretching involved the standing hamstring stretch or the sitting toe touch stretch. For the standing hamstring stretch, participants stood with the involved leg elevated on a chair or stool. While standing tall, maintaining the neutral spine posture, and keeping the involved knee in full extension, subjects were instructed to lean forward hinging at the hips until moderate stretch discomfort was felt in the hamstrings [29]. For the sitting toe touch stretch, participants sat with both legs extended while their trunk remained straight, and then, they extended their hand forward towards their feet as far as possible while keeping the knees straight. Knee extensor self-stretching involved the standing quadriceps stretch or the prone quadriceps stretch. For the standing quadriceps stretch, participants stood on one side of the healthy side, the side of the hand was used to hold the table or the wall and the other hand grasped the affected side of the ankleand stretched backwards until the affected quadriceps or knee joint had a feeling of soreness. For the prone quadriceps stretch, participants lay prone while flexing their involved knee and pulling the foot toward the back with their ipsilateral hand. If a participant was unable to do it by themself, passive stretching was used to provide assistance. For the gluteus stretch, the participant stood beside the bed or table, kept the trunk parallel to the bed or table, bent the knee joint of the affected side, placed it flat on the bed, stood with the healthy side leg and extended it backward, and leaned forward until they feltthat the hip of the affected side was extended. For the adductor stretch, the participant keptthe body upright with hands on the hips. He bent the knee of the healthy side so that the knee was directly above the foot, kept the affected leg straight, and touched the foot. He slowly moved the body's center of gravity to the healthy side until he could feel that the adductor muscle on the affected side extend. Those who had difficulty in completing the task could sit in a supported position. For the posterior calf stretching, participants stood with both feet forward facing towards a wall with hands resting on the wall for support; then they placed the affected foot back into a lunge position and felt a stretch in the calf muscle located in the back of the lower leg by increasing the bending of the front knee. Participants were instructed to ensure that they kept the back heel flat on the floor.

The stretching of the patient depended on the muscle with the trigger point, that is, when a certain muscle had a trigger point, the corresponding muscle stretch was performed.The above static stretching was maintained for 1 min and repeated three times per day. The extent of stretching is suitable for tightness, if there was discomfort such as numbness or pain, the patient needed to reduce the extent and time of stretching. Even after terminating the dry needling or oral medicine therapies, all patients would continually conduct it.

## The diclofenac group (DG)

### Oral diclofenac and stretching

Diclofenac sodium double-release enteric-coated capsules (manufactured by Temmler Ireland Ltd, Killorglin, Co., Kerry, Ireland), each containing 75 mg of diclofenac sodium, were taken orally 75 mg a day before a meal without chewing, together with a glass of water every time and once a day for 6 weeks as a treatment course combined with self-stretching at home (same as that in the DNG).

### Statistical analysis

All data were analyzed using the Statistical Package for Social Sciences (SPSS) V.22.0. The mean and SD for each quantitative variable were calculated after confirming normal distribution of the data using the Shapiro–Wilk test. The independent t-test was used to compare the differences in the underlying data of the subjects included. Two-way repeated measures analysis of variancewith a post-hoc Bonferroni correction was used, and the level of significance was set at 5% (*p* < 0.05).

## Results

### CONSORT flow diagram of the procedures

A flow chart of the procedures involving study participation and follow-up is illustrated in Fig. 1. A total of 166 patients were assessed for eligibility. A total of 68 patients were excluded (56 did not meet the inclusion criteria and 12 patients declined to participate in the study), thus resulting in a total of 98 patients who were included in the study. Furthermore, 49 patients were randomized to the DNG and 49 patients were randomized to the DG. During the 6-week treatment, four participants in the DNG and three participants of the DG discontinued treatment, two participants in the DNG and five of the DG withdrew due to lack of effect, three participants in the DG withdrew due to adverse event, and one participant in the DG withdrew for personal reasons. At the 6-month follow-up, three participants dropped out because of losing contact (1 in the DNG and 2 in the DG). Finally, the analyses were performed in 77 patients who completed the study (42 in the DNG and 35 in the DG).

### Baseline characteristics

The baseline demographic data of the participants are presented in Table 1. There was no significant difference in gender, age, height,weight, body mass index (BMI), duration of pain, and the severity of knee involvement on the X-ray between the two groups. No statistically significant difference was observed in the baseline values of study outcomes.

**Table 1:** Baseline characteristics (mean ± SD)

Characteristics	DNG (*n* = 42)	DG (*n* = 35)	*P* value
gender (Male / Female)	13/29	12/23	
Age (years)	74.61 ± 6.43	75.39 ± 5.77	0.14
Height (cm)	161.46 ± 7.56	163.46 ± 7.35	0.55
Weight (kg)	68.41 ± 7.26	65.13 ± 8.95	0.31
Body Mass Index (kg/m2)	26.10 ± 3.41	24.49 ± 3.29	0.76
Duration of pain (mo)	59.41 ± 17.33	65.62 ± 15.05	0.37
Kellgren-Lawrence OA grade n (%)			
Grade 2	20 (47.6%)	17 (48.6)	
Grade 3	17 (40.5%)	15 (42.9)	
Grade 4	5 (11.9%)	3 (9.6)	

Data are presented as mean ± SD

*DNG* Dry Needling group, *DG* Diclofenac group

### Prevalence of latent and active MTrPs

The prevalence of latent and active MTrPs are presented in Table 2. The prevalence of latent MTrPs in DNG was estimated to be 50.0%, 45%, 64%, 42.9%, 40.5%, 16.7%, 23.8%, 54.8%, 28.6%, 42.9%, 71.4%, and47.6%forrectus femoris, vastus medialis, vastus lateralis, tensor fasciae latae, hip adductors, gluteus maximus, gluteus medius, biceps femoris, semitendinosus-semimembranosus, popliteus, gastrocnemius, and soleus muscle, respectively. The prevalence of active MTrPs in DNG was estimated to be 38.1%,31%,54.8%,21.4%,23.8%, 7.1%, 16.7%,47.6%,11.9%,28.6%, 50%, and 33.3% forrectus femoris, vastus medialis, vastus lateralis, tensor fasciae latae, hip adductors, gluteus maximus, gluteus medius, biceps femoris, semitendinosus-semimembranosus, popliteus, gastrocnemius, and soleus muscle, respectively.

**Table 2:** Prevalence of active and latent MTrPs in patients with knee osteoarthritis (KOA)

Muscle	DNG -LMTrPs		DNG -AMTrPs		DG-LMTrPs		DG-AMTrPs	
	Number (42)	Percentage(%)	Number (42)	Percentage (%)	Number (35)	Percentage (%)	Number (35)	Percentage (%)
rectus femoris	21	50%	16	38.1%	19	54.3%	14	40%
vastus medialis	19	45%	13	31%	18	51.4%	12	34.2%
vastus lateralis	27	64%	23	54.8%	20	57.1%	17	48.6%
tensor fasciae latae	15	42.9%	9	21.4%	12	34.3%	7	20%
hip adductors	17	40.5%	10	23.8%	10	28.6%	6	17.1%
gluteus maximus	7	16.7%	3	7.1%	4	11.4%	2	5.7%
gluteus medius	10	23.8%	7	16.7%	11	31.4%	6	17.1%
biceps femoris	23	54.8%	20	47.6%	18	51.4%	14	40%
Semitendinosus- semimembranosus	12	28.6%	5	11.9%	7	20%	3	8.6%
popliteus	18	42.9%	12	28.6%	12	34.3%	8	22.6%
gastrocnemius	30	71.4%	21	50%	23	65.7%	16	45.7%
soleus muscle	20	47.6%	14	33.3%	17	48.6%	12	34.3%

Abbreviations: *DNG* dry needling group, *DG* Diclofenac group, *LMTrPs* latent myofascial trigger point, *AMTrPs* active myofascial trigger points

The prevalence of latent MTrPs in DG was estimated to be 54.3%, 51.4%, 57.1%, 34.3%, 28.6%, 11.4%, 31.4%, 51.4%, 20%, 34.3%, 65.7%,and 48.6%forrectus femoris, vastus medialis, vastus lateralis, tensor fasciae latae, hip adductors, gluteus maximus, gluteus medius, biceps femoris, semitendinosus-semimembranosus, popliteus, gastrocnemius, and soleus muscle, respectively. The prevalence of active MTrPs in DG was estimated to be 40%, 34.2%, 48.6%, 20%, 17.1%, 5.7%, 17.1%, 40%, 8.6%, 22.6%, 45.7%, and 34.3% forrectus femoris, vastus medialis, vastus lateralis, tensor fasciae latae, hip adductors, gluteus maximus, gluteus medius, biceps femoris, semitendinosus-semimembranosus, popliteus, gastrocnemius, and soleus muscle, respectively.

### NPRS, WOMAC, and ROM

The results for treatment effects on symptom outcomes are shown in Table 3. In the DNG, the NPRS score was significantly decreased at the 6-week and 6-month follow-up compared to that at the pre-treatment timepoint (*P* < 0.05). The NPRS score in the DG showed a significant decrease after the 3 week and the 6-month follow-ups compared with that at the pre-treatment timepoint (*p* < 0.05). However, there was a significant increase in the NPRS score at the 6-month follow-up compared with that at the 6-week timepoint (*p* > 0.05). Comparison between groups showed that there was no significant difference between the DNG and the DG before treatment (*p* > 0.05), and the DNG showed significantly lower scores than the DG after 6 weeks and 6 months of follow-up (*p* < 0.05).

**Table 3:** Treatment effects on symptom outcomes

Group(n)	PT	6 W	6 M	Between group(DNG vs. DG)								Time × Group
				**PT**			**6 W**			**6 M**		
	**(Mean ± SD)**	**(Mean ± SD)**	**(Mean ± SD)**	**Effect size**		***P*-value**	**Effect size**		***P*-value**	**Effect size**	***P*-value**	**Effect size**
				**(95% CI)**			**(95% CI)**			**(95% CI)**		***P*-value**
**NPRS**				0.009		0.41	0.133	0.001		0.17	< 0.001	0.136
				( -0.42, 1.02)			(-1.496, -0.404)			(-1.835, -0.615)		0.001
DNG(*n* = 42)	6.15 ± 1.48	2.10 ± 1.28^*△^	2.45 ± 1.22^*△^									
DG(*n* = 35)	5.85 ± 1.75	3.05 ± 1.18^*^	3.68 ± 1.51^*#^									
**WOMAC-pain**				0.042	0.068		0.081		0.01	0.165	< 0.001	0.199
				(-0.068, 1.868)			(-1.626, -0.224)			(-2.035,-0.665)		< 0.001
DNG(*n* = 42)	7.85 ± 2.18	2.6 ± 1.63^*△^	2.73 ± 1.38^*△^									
DG(*n* = 35)	6.95 ± 2.17	3.53 ± 1.52^*^	4.08 ± 1.69^*^									
**WOMAC-stiffness**				0.003	0.640		0.097		0.005	0.175	< 0.001	0.094
				(-0.486, 0.786)			(-1.096, -0.204)			(-1.415, -0.485)		0.006
DNG(*n* = 42)	3.53 ± 1.50	1.38 ± 1.13^*△^	1.63 ± 1.19^*△^									
DG(*n* = 35)	3.38 ± 1.35	2.03 ± 0.86^*^	2.58 ± 0.87^*^									
**WOMAC-function**				0.006	0.49		0.254		< 0.001	0.315	< 0.001	0.174
				(-2.224, 4.644)			(-10.957, -4.725)			(-10.957, -5.493)		< 0.001
DNG(*n* = 42)	28.05 ± 8.18	9.13 ± 5.82^*△^	11.45 ± 5.38^*△^									
DG(*n* = 35)	26.85 ± 7.27	16.83 ± 7.45^*^	19.68 ± 6.81^*^									
**WOMAC-total**				0.018	0.242		0.296		< 0.001	0.403	< 0.001	0.251
				(-1.546, 6.046)			(-12.497, -6.053)			(-13.411, -7.639)		< 0.001
DNG(*n* = 42)	39.43 ± 8.96	13.1 ± 6.53^*△^	15.80 ± 5.70^*△^									
DG(*n* = 35)	37.18 ± 8.07	22.38 ± 7.88^*^	26.33 ± 7.18^*#^									
**ROM**				0.003	0.626		0.060		0.029			0.163
				(-8.245, 4.995)			(0.792, 14.458)					< 0.001
DNG(*n* = 42)	96.63 ± 15	114.50 ± 15.31^*△^										
DG(*n* = 35)	98.25 ± 14.74	106.88 ± 15.39^*^										

*DNG* Dry Needling group, *DG* Diclofenac group, *PT* Pre-treatment, *6 W* 6 weeks after treatment, *6 M* 6 months after treatment

^*^Compared with the pre-treatment within-group *p* < 0.05

^#^compared with 6 weeks after treatment within-group *p* < 0.05

^△^compared with the control group between-group *p* < 0.05

The WOMAC (pain, stiffness, function, total) scores in the DNG and DG were significantly decreased at the 6-week and 6-month follow-ups compared to those at the pre-treatment time point (*P* < 0.05). However, in the DG, there was a significant increase in the WOMAC (total) score at the 6-month follow-up compared with that at the 6-week timepoint (*p* > 0.05). Comparison between groups showed that there was no significant difference between the DNG and the DG before treatment (*p* > 0.05), and the DNG showed significantly lower scores than the DG after 6 weeks and 6 months of follow-up (*p* < 0.05).

With respect to active ROM of knee flexion, there was a significant increase in the DNG and DG at 6 weeks of treatment compared to that at the pre-treatment time point (*P* < 0.05). Comparison between groups showed that there was no significant difference between the DNG and the DG before treatment (*p* > 0.05), and the DNG showed a significantly increased ROM than the DG after 6 weeks of treatment (*p* < 0.05).

### Adverse events

Common dry needling treatment-related adverse events include subcutaneous hematoma, continuous post-dry needling pain, and dizziness caused by hypoglycemia. Common adverse events related to oral diclofenac sodium are gastrointestinal adverse events, renal-function adverse events, and hepatic-function and cardiovascular adverse events. According to the previous literature, all of these adverse drug reactions are predictable. During the 6 weeks’ treatment period, two patients in the DNG dropped out of the trial because of continuous post-dry needling pain, three patients dropped out of the trial because of indigestion (*n* = 2) and abdominal pain (*n* = 1), and no serious adverse drug reactions were found in this clinical trial.

## Discussion

The current study investigated the effect of treatment with dry needling latent and active MTrPs combined with knee muscle stretching, and it compared that effect with the effect of treatment with oral diclofenac and knee muscle stretching. After treatments, both the groups showed a good effect in knee pain, function, and ROM, However, the DNG showed significantly better results than the DG. Especially in the results ofthe 6-month follow-up, the results of the DNGweresuperior tothose of the DG.

Differentfrompreviousstudiesthat focused on dry needling active trigger points in the treatment of KOA, in this study, in addition to dry needling active trigger points, latent trigger points were also included, and there were several reasons why the latent trigger points dry needle were included. First, latent trigger points have a high prevalence in KOA. The research of Sánchez et al. [10] showed that the prevalence of latent MTrPs varied from 11 to 50% in various muscles of patients with mild to moderate painful KOA.In contrast, the tensor fasciae latae showed the highest prevalence of latent MTrPs (50%). Our study showed a high prevalence of latent trigger points in rectus femoris, vastus lateralis, biceps femoris, and gastrocnemius, all of which wereover 50%, and gastrocnemius wasthe highest(68.8%). The prevalence of latent trigger points in other muscles was also higher than that in Sánchez et al. The reason may be that the participants of the two studies were different. Most of the participants in Sánchez's study were mild to moderate(Kellgren and Lawrence scale between 1–3). However, the subjects included in this study were mostly moderate to severe (Kellgren and Lawrence scale between 2–4). Therefore, it is possible that the more severe the symptoms of KOA patients, the higher the prevalence of latent trigger points.Second, compared with the activate trigger points, latent MTrPs do not produce spontaneous and recognizable pain under stimulation, however, latent MTrPs play a role in limiting the range of motion, reducing muscle strength, accelerating fatigability, and altering muscle contraction patterns [30]. Restricted joint ROM is commonly observed in patients with latent MTPs. The number of latent MTPs has been reported to be negatively correlated with the baseline ROM [31]. Baraja-Vegas [32] observed an increased stiffness in individuals with latent MTPs,andthis increased muscle stiffness alters muscle contractile properties, restricts joint range of motion, provokes muscle weakness, and accelerates fatigability. In addition, Ge et al. [33] found that this motor dysfunction may result in incoherent muscle activation of synergists inducing impaired motor control strategies.Third, in the past, latent trigger points were mostly included in the study of healthy subjects or asymptomatic subject [34–36].

However, in recent years, latent trigger points have received attention and have been included in the treatment of various skeletal muscle pain. Calvo Lobo's research showed that dry needling intervention of the latent MTrPwasassociated with the key active MTrP of the infraspinatus reduces pain intensity in the short term in older adults with nonspecific shoulder pain [37]. The results of Sánchez-Infante's study showed that the application of one session of DN over LTrP decreased the pressure pain, dynamic stiffness, and muscle stiffness values at 72 h after treatment [38].Another study by Sánchez-Infante showed that one session of DN intervention in latent trigger points of the upper trapezius muscle reduced muscle stiffness and the pressure pain threshold for the dry needling group compared to the sham dry needling group [30]. Latent trigger points have alsobeenincluded in studies of nonspecific chronic low back pain [39]. However, in the trigger point treatment of KOA, no latent trigger point has been involved.

Currently, for the treatment of KOA, the active trigger point is primarily involved, and the active trigger point of quadriceps femoris is primarily included because the referred pain of the quadriceps femoris is near the patellofemoral joint. When activated, there is pain in the patellofemoral joint. Patellofemoral Pain Syndrome(PFPS), as the early lesion of OA, often achieves positive effects by needling the active trigger point of the quadriceps femoris [40, 41]. However, only the active trigger point was included in the treatment of KOA, which cannot achieve a positive effect [18, 42]. Therefore,latent trigger points may also need to be included in the treatment of KOA, and the reason may be because active and latent MTrPs, as a bundle spasm of muscle fibers, can cause muscle force imbalance, which generates an uneven stress to a certain location in the joint and accelerates cartilage destruction [43]. If it is not treated in time, eventually, this can result in articular dysfunction from synovial stagnation, hypoxia, synovial hyperplasia, biochemical derangements, angiogenesis, effusion, bone remodeling, and inflammation [44].In this study, the muscles involved included the quadriceps, the tensor fasciae latae, the hip adductors, the hip abductors, the hamstrings, and the triceps calf and popliteus muscles. These muscles affect the function and biomechanics of the knee joint. Once the trigger points (latent or active) appears, it may lead to abnormal cartilage load through muscle weakness or tightness, and this may further exacerbate the degenerative process of the knee joint [45, 46].

The effects of the included muscles on joint mechanics are as follows:

- (1). Quadriceps tightness can lead to an increased compression force of the patellofemoral joint [47, 48], or because themedial and lateral components of the quadriceps exert different mediolateral forces at the patella, their unbalancemay alter the pressure distribution across the patellofemoral joint and patellar kinematics [40, 49].
- (2). The tensor the fascia lata is connected to the patellofemoral through the iliotibial band (ITB) and the sateral patellar retinaculum. The increased tension of the tensor fascia lata increases thepatellar lateral translation and tilt and increases thelateral cartilage pressure [50, 51].
- (3). The hamstrings, apart frombeing the primary mechanism of knee flexion, also protect the knee from eccentric contraction during the support phase. These both function to cushion the joint and to generate limb deceleration during gait [52]. In addition, as an antagonist of the quadriceps, tightness of the hamstrings may require higher quadriceps force production or cause slight knee flexion, resulting in increased patellofemoral joint reaction forces [53, 54].
- (4). Inanopen-chain, the gastrocnemius muscle can bend the knee joint and ankle plantar flexion, and the soleus muscle can also bend the ankle plantar flexion. However, inaclosed-chain, the gastrocnemius muscle and soleus muscle pull the lower end of the femur and the lower leg backward to straighten the knee joint [55]. In fact,thegastrocnemius and quadriceps femoris have a co-activation effect when squatting up. However, when the soleus muscle is insufficient, the quadriceps femoris has to use more force to stabilize the knee joint, resulting in strain ofthequadriceps femoris [56].The primary role of the popliteus muscle is to internally rotate the tibia in relation to the femur in open-chain and stabilization of the external rotation of the femur in relation to the tibia in closed chain situations, and the popliteus muscle tightness will cause difficulty in stretching the knee joint [57, 58].
- (5). The gluteus medius is theprimary abductor of the hip. During walking, weak hip abductors of the stance limb produce a pelvic drop on the swing limb and stance knee varus angulation that shifts the line of gravity (LOG) away from the stance knee. This LOG shift increases the knee adduction moment and the medial joint compressive forces, leading to progressive degeneration. The hip adductor muscles may eccentrically counteract the varus angulation of the knee and consequently might unload the medial tibiofemoral joint [59]. A systematic review identified moderate quality evidence that suggested substantial hip abductor and adductor muscle weakness in people with knee OA [60].
- (6). Hip external rotation (the posterior fibers of the gluteus medius and maximus muscles) act eccentrically to control the movements of hip medial rotation during activities with body weight support.The weakness of hip external rotation leads to the internal rotation of the femur, and this contributes to increased lateral forces acting on the patella and greater stress on the lateral patellofemoral joint [61, 62].

Previous studies have reported that ROM of the knee joint is decreased gradually in individuals with KOA [63]. It is essential to restore normal length and flexibility to the muscles. Thus, stretching exercises are an effective complementary therapy and play an important role in the treatment of patients with KOA [16, 64]. The stretching technique for muscles improved pain ratings, joint stiffness, function, and ROM, and specifically, stretching therapy for the surrounding muscles of the knee can improve muscle flexibility and correct muscle imbalance so as to decrease the stress concentration in the knee. However, one should be careful of achieving this by direct stretching exercises when a muscle is still in pain and spasm. Direct stretching may cause more pain and more spasm in the painful muscle [65]. Therefore, stretching is appropriate after pain relief. In this study, dry needling and oral drugs in the DNG and DG groups significantly reduced pain, and the ROM of both groups significantly improved through treatment combined with stretching. However, the ROM of the DNG group was significantly better than that of the DG group. Similar to this study, previous research has reported improved joint ROM follwing dry needling trigger points [66, 67]. The observed changes in joint ROM could be associated with a relaxation of the MTrPs [68]. The reason might be that TrPS can change the structure of the muscle by increasing the stiffness in muscle cells and tissues, and the dry needling can release the contracture nodule of the trigger points, thus decreasing resistance when stretching the muscle [69, 70].

Traditionally, clinicians have relied heavily on the use of NSAIDs, including diclofenac sodium to treat the symptoms of KOA, such as inflammatory pain and joint stiffness. Diclofenac is a proven, commonly prescribed nonsteroidal anti-inflammatory drug (NSAID) that has analgesic, anti-inflammatory, and antipyretic properties and has been shown to be effective in treating a variety of acute and chronic pain and inflammatory conditions.Despite its side effects, diclofenac sodium still remains the drug of choice [71]. Clinical studies have repeatedly shown that NSAIDs can alleviate pain and improve function in KOA patients [72]. In terms of the NPRS and WOMAC function, DNG and DG improved significantly in 6-week and 6-month follow-ups compared with pre-treatment. However, the DNG showed advantages in the NPRS and WOMAC function whether in the 6-week or 6-month follow-up. Moreover, DG showed significant regression at the 6-month follow-up compared with the 6-week in NPRs and WOMAC- total. However, DNG showed no significant difference between 6-months follow-up and 6-week. The advantages of dry needling trigger points may be that compared with oral diclofenac, dry acupuncture trigger point can not only alleviate pain, but also restore the mechanical imbalance of the skeletal muscle around the knee joint caused by the trigger point, thus improving the clinical symptoms of knee osteoarthritis.

## Limitations

This experiment has certain limitations,one of which is the lack of supervision of the patient’s self-stretching. To cover the ethical and feasibility considerations, this study compared two treatment groups, but no placebo group was used in this experiment. Because current studies have shown no correlation between pain and osteoarthritis classification, this experiment did not analyze and statistics according to the classification of osteoarthritis. Most of our follow-ups were conducted by telephone because the patient was too old and has limited physical mobility. Therefore, no ROM was measured. However, the patient may not have completed the recommended exercise. Finally, previous studies primarily focused on active trigger points, that is, inactivate active trigger points to latent trigger points. However, how to evaluate the effect of dry needling on latent trigger points requires further research to investigate this important issue. The pressure pain threshold (PPT) may have important value for the evaluation of latent trigger points; however, there was no record due to a lack of appropriate instruments.

## Conclusions

Compared with the diclofenac group, the dry needling group manifested persisting superiority and clinically relevant benefits for at least 6 months in patients with KOA. The benefits included a decreased pain intensity and disability as well as knee ROM.

## Data Availability

The datasets generated and analyzed during the current study are available from the corresponding author on reasonable request.

## Abbreviations

MTrPs: Myofascial trigger points
KOA: Knee osteoarthritis
ROM: Range of motion
NPRS: Numeric Pain Rating Scale
WOMAC: Western Ontario and McMaster Universities Osteoarthritis Index
DNG: Dry needling group
DG: Diclofenac group
NSAIDs: Non-steroidal anti-inflammatory drugs
TKA: Total knee arthroplasty
RCT: Randomized controlled trial
SPSS: Statistical Package for Social Sciences
BMI: Body mass index
ITB: Iliotibial band
LOG: Line of gravity
PPT: P ressure pain threshold
